# Oral eltrombopag versus subcutaneous recombinant human thrombopoietin for promoting platelet engraftment after allogeneic stem cell transplantation: A prospective, non‐inferiority, randomized controlled trial

**DOI:** 10.1002/hon.3017

**Published:** 2022-05-18

**Authors:** Bingbing Wen, Xiaohan Zhang, Shiyu Chen, Jingchao Fan, Sitian Yang, Yun Cai, Pengcheng Wang, Qiaoxia Zhang, Qingli Gu, Xin Du

**Affiliations:** ^1^ Department of Hematology The First Affiliated Hospital of Shenzhen University Shenzhen Second People's Hospital Shenzhen China

**Keywords:** allogeneic stem cell transplantation, eltrombopag, platelet engraftment, recombinant human thrombopoietin, rhTPO

## Abstract

Delayed platelet engraftment (DPE) is associated with poor survival and increased transplantation‐related mortality after allogeneic hematopoietic stem cell transplantation (allo‐HSCT). Therefore, treatments are needed to improve platelet engraftment and prevent DPE. We performed a phase three, non‐inferior, randomized controlled study of eltrombopag or recombinant human thrombopoietin (rhTPO) to promot platelet engraftment after allo‐HSCT. Candidates for allo‐HSCT were randomly assigned to receive oral eltrombopag (50 mg daily) or **subcutaneous** rhTPO (15000U daily) from the first‐day post‐transplantation. The primary endpoint was the cumulative numbers of platelet engraftment (platelet recovery ≥20 × 10^9^/L, without transfusion, for seven consecutive days) on day 60 after transplantation. We performed intention‐to‐treat analyses with a non‐inferior margin of −15%. A total of 92 participants underwent randomization. 44 and 48 patients were randomized to the eltrombopag and rhTPO groups, respectively. The median duration of follow‐up was 360 days (range: 12–960 days). The cumulative incidence of platelet engraftment on day 60 after transplantation in eltrombopag group was 86.4% (38/44) compared with 85.4% (41/48) in the rhTPO group (absolute risk difference [ARD] 1%, one‐sided lower limit of 95% confidence interval [CI] −13.28%, P_non‐inferirioty_ = 0.014). The rate of DPE in the eltrombopag group was 6.8% (3/44) compared with 12.5% (6/48) in the rhTPO group (ARD ‐5.7%, one‐sided higher limit of 95% CI 6.28%, P_non‐inferirioty_ = 0.063). Approximately, three‐fourths of non‐hematologic adverse events were not observed in the eltrombopag group but three patients (3/48, 6%) experienecd them in the rhTPO group. In addition, platelet transfusions unite from day 0 to day 21, or from day 22 to day 60, progression‐free survival, overall survival were not significantly different between both groups. Eltrombopag was non‐inferior to rhTPO in promoting platelet engraftment post allo‐HSCT for patients with hematological malignancy. Oral eltrombopag was more convenient for patients than **subcutaneous** rhTPO (NCT03515096).

## 1 INTRODUCTION

Allogeneic hematopoietic stem cell transplantation is an important method for hematological malignancy.^1^, ^2^ Delayed platelet engraftment (DPE), occurring in 5%–37%, is a common complication post transplantation‐related mortality after allogeneic hematopoietic stem cell transplantation (allo‐HSCT).^3^ A study by Kanda et al. showed that 69% of patients receiving myeloablative conditioning were reported with cumulative events of platelet recovery within 60 days^4^ Platelet and immune recovery are strongly associated with treatment‐related mortality and overall survival (OS).^5^, ^6^, ^7^ However, it is lack of reliable and effective methods to improve platelet recovery and prevent DPE. Intravenous immunoglobulin, corticosteroids, repeated platelet transfusions and rituxan remain important methods^8^ and connected with a higher risk of costs and infection, and impact on patients' lives.

Thrombopoietin (TPO), a cytokine that stimulates stem cells to differentiate into megakaryocytes, promotes megakaryocyte proliferation and to drive thrombopoiesis.^9^, ^10^, ^11^, ^12^, ^13^, ^14^ Studies have showed TPO is an important physiological cytokine that stimulates platelet production.^11^, ^12^, ^13^, ^14^, ^15^, ^16^ Many studies have been demonstrated the safety of recombinant human TPO (eltrombopag or recombinant human thrombopoietin (rhTPO)).^17^, ^18^, ^19^ Ting‐ting Han et. al. showed that the cumulative events of platelet engraftment on day 60 in rhTPO group was statistically higher than that in control group after transplantation (*P* = 0.041).^20^ However, the response of treatment with rhTPO was only 45.8% for patients with DPE after allo‐HSCT,^21^ and subcutaneous rhTPO was not conveninent for patients.

Eltrombopag, a TPO receptor (TPO‐R) agonists that promotes platelet production, has been supported for the treatment of idiopathic thrombocytopenia purpura.^22^, ^23^ As an oral non‐peptide agonist, it binds to TPO‐R site to improve the differentiation and proliferation of megakaryocytes. The safety and efficacy of eltrombopag have been demonstrated in severe aplastic anemia^24^ and thrombocytopenia associated with hepatitis C infection^25^ and severe aplastic anemia.^24^ Luigi J. et. al. Showed that eltrombopag mediates proinflammatory cytokine interferon‐γ (IFNγ) to maintain human hematopoietic stem and progenitor cells under inflammatory conditions, which suggests a possible immune regulatory mechanism to enhance platelet engraftment after hematopoietic stem cell transplantation (HSCT).^26^ The efficacy of eltrombopag for the treatment of thrombocytopenia post HSCT has been published in several case reports.^27^, ^28^, ^29^ Besides, Yifang Yao et.al confirmed that pooled response rate of treatment with TPO‐R was higher than that of rhTPO on thrombocytopenia after HSCT (73% vs. 27.8%).^30^ However, efficacy of eltrombopag versus rhTPO for improving platelet recovery after allo‐HSCT has not yet been assessed.

We designed the randomized controlled trial to evaluate whether oral eltrombopag (50 mg, daily) was non‐inferior to subcutaneous rhTPO (15000U, daily) in promoting platelet engraftment post allo‐HSCT for patients with hematological malignancy. The number of platelet transfusions, transplant‐associated complications, and Adverse events (AEs) were also assessed in this study.

## 2 MATERIALS AND METHODS

### 2.1 Trial design and patients

This phase three, single‐center, non‐inferiority, randomized controlled study was performed at Second People's Hospital of Shenzhen, China. Key eligibility criteria, including an age ≥18 years, a confirmed diagnosis of hematological malignancy who were prepared to accept allo‐HSCT, and an Eastern Cooperative Oncology Group performance‐status score <2 were eligible for inclusion. Previous receipt of any eltrobompag or rhPTO before allo‐HSCT was excluded. Patients who have a history of severe lung disease or congestive heart failure were also excluded from the study. Molecular mutations were detected at a central laboratory.

The Institutional Review Board of the Second People's Hospital of Shenzhen approved this study, and this trial was designed based on the Declaration of Helsinki and Good Clinical Practice Guidelines. All participants were required to provid informed consents before this study (NCT03515096).

### 2.2 Randomization and intervention

Participants were randomly assigned at a rate of 1:1 with a web‐based, electronic system. An independent statistician generated the allocation sequence based on computerized random number and concealed. Eligible patients were randomized to receive either oral eltrombopag (Beijing Novartis Pharma Ltd., Beijing, China) (50 mg, daily) or subcutaneous rhTPO (Shenyang Sunshine Pharmaceutical Co., Ltd., Shenyang, China) (15000U, daily) from the first‐day post‐transplantation. When platelet count was ≥50 × 10^9^/L for three consecutive days, rhTPO or eltrombopag was discontinued.

All patients for myeloablative conditioning accepted a modified BuCy with or without an antihuman thymocyte immunoglobulin (ATG) regimen,^31^, ^32^, ^33^ including an busulfan (0.8 mg/kg in 12 doses, days 8, 7, 6), cytosine arabinoside (4 g/m^2^/day, days 10, 9), semustine (250 mg/m^2^, day 3), cyclophosphamide (1.8 g/m^2^/day, days 5, 4), and ATG (2.5 mg/kg/day, from day 5 to day 2).

All donors were required to receive rh‐G‐CSF (5 μg/kg for 5 or 6 days, daily). Peripheral blood progenitor and bone marrow (BM) cells were collected on day 5 and day 4, respectively. Then all participants received peripheral and BM blood of donors (5 μg/kg/d, from day 6 after transplantation to neutrophil count ≥0.5 × 10^9^/L, for three consecutive days). In addition, Mycophenolate mofetil with short‐term methotrexate and cyclosporine A were administered as prophylaxis for graft‐versus‐host disease (GVHD).^31^, ^33^ Supportive care measures were provided to given to all patients, including transfusions, growth factor support administered per institutional standards and antimicrobial agents.

### 2.3 End points and assessments

The primary endpoint was the cumulative numbers of platelet engraftment (platelet recovery ≥20 × 10^9^/L, without transfusion, for seven consecutive days) on day 60 after transplantation. The secondary endpoints included the number of platelet transfusions from day 0 to day 21 or from day 22 to day 60, Progression‐free survival (PFS), OS and safety. Safety was assessed according to the incidence of laboratory abnormalities, transplant‐associated complications and bleeding events. Bleeding events were assessed based on a predefined bleeding scoring system.^34^ AEs included laboratory abnormalities and clinical symptoms^17^ within 100 days after transplantation. In addition, abnormalities in renal, hepatic, and coagulative functions were included as laboratory abnormalities based on the Common Terminology Criteria for Adverse Events version 4.0.

Complete blood counts and chemical analyses, including total bilirubin, direct bilirubin, for glutamic‐pyruvic transaminase, Scr, blood urea nitrogen, and glutamic‐oxaloacetic transaminase, for patients were assessed three times each week within 30 days after transplantation and weekly thereafter until day 100. Epstein‐Barr virus (EBV) and cytomegalovirus (CMV) DNA tests were conducted weekly until day 100. The day of transplantation was day 0.

The date of platelet engraftment was defined as a platelet count ≥20 × 10^9^/L without transfusion for seven consecutive days. DPE was defined as a platelet count <20 × 10^9^/L by day 60 after transplantation.^20^, ^35^ Secondary post‐transplant thrombocytopenia (SPT) was defined as a platelet count decreased <20 × 10^9^/L post first platelet engraftment.^20^ Idiopathic SPT was defined as SPT in absence of relapse or sepsis, within the first 100 days after transplantation.^36^, ^37^ Based on the Chinese guidelines, platelet transfusion was performed in patients with an important bleeding risk or a platelet count <20 × 10^9^/L. A volume of approximately 200 ml and 2.5 × 10^11^ platelets were included in each platelete unit.

Graft‐versus‐host disease was defined according to the published criteria.^38^ PFS was defined as the duration from day 0 to relapse or last follow‐up. OS was defined as the duration from day 0 to death or last follow‐up. Immune recovery using flow cytometry was conducted on day 30, according to the number of CD3+, CD4+, CD8+, and CD19+ cells.

### 2.4 Statistical analysis

The clinical data cut‐off date was 20 December 2021. The population was evaluated using the intention‐to‐treat (ITT) method, including all 92 patients, and analyzed for efficacy and safety in this trial. Summary statistics, including mean, proportion, range, and median, were calculated for patient characteristics, platelet transfusion units and AEs. Categorical and continuous variables were analyzsd by χ2 test and Mann–Whitney *U* test, respectively. PFS and OS were analyzed with Kaplan‐Meier method, and log‐rank test was used to compare for each group. 95% CIs and hazard ratios (HRs) and were performed with a stratified Cox proportional hazard regression model. Confidence intervals around the risk difference and *p* values for non‐inferiority were calculated using SAS studio with program code (https://welcome.oda.sas.com/home)^39^ between two groups for the cumulative incidence of platelet engraftment on day 60

A design of non‐inferiority was conducted because this goal was to evaluate an alternative treatment option to eltrombopag with rhTPO. Study by Ting‐ting et. al. study showed that cumulative incidence of platelet engraftment on day 60 after transplantation in rhTPO group was 91.7% (55/60, 95%CI: 84.7%–98.7%). The margin of non‐inferiority was performed at a 15%. When the difference of the lower bound of one‐sided 95% CI didn't exceed −15%, it could be proved that eltrombopag was non‐inferior to rhTPO. With one‐sided level of 0.025% and 80% power, 98 patients were estimated for ITT analysis, supposing an expected response of 90% in rhTPO group and 92% in eltrombopag group.^20^ The non‐inferiority margin for platelet engraftment of eltrombopag versus rhTPO was defined using a 20% retention of the lower bound (84∙7%) of the 95% CI of the Ting‐ting et. al. trial. When non‐inferiority was demonstrated, a post‐hoc superiority analysis was then performed (Stata version 14.1 and SAS version 9.4).

## 3 RESULTS

### 3.1 Characteristics of patients

**FIGURE 1:**
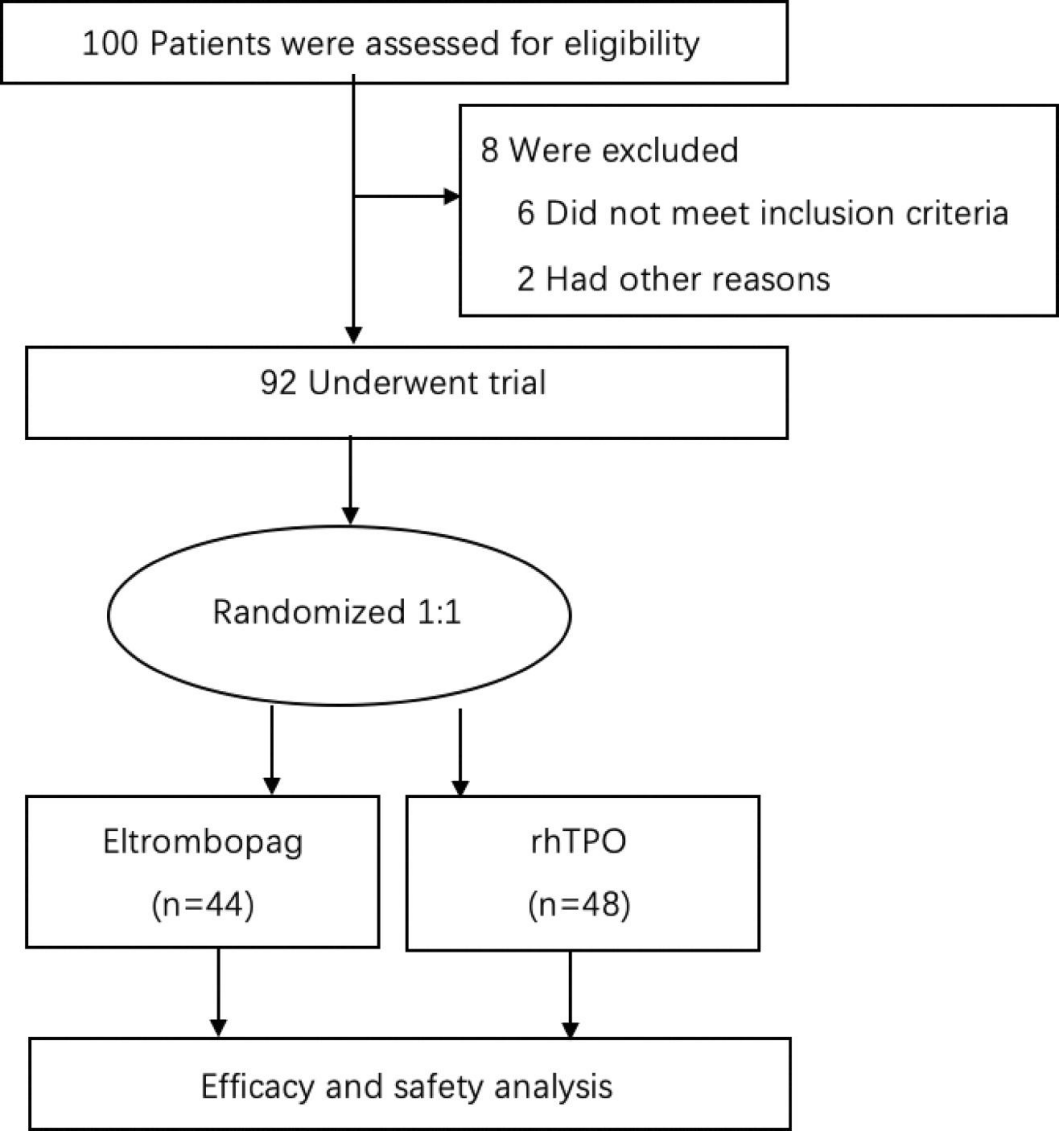
Randomization and treatment

Between July 2018 and December 2021, a total of 100 patients underwent screening, and 92 patients were randomized at a 1:1 ratio in our hospital. 44 patients were randomized to eltrombopag group and 48 were assigned to rhTPO group (Figure 1). The median age in the eltrombopag and rhTPO groups was 36 years (range, 18–54 years) and 37 years (range, 18–57 years), respectively (*P* = 0.997). 66% and 52% of patients were male in eltrobompag and rhTPO groups, respectively (*P* = 0.179). Patients with acute myeloid leukemia in eltrobompag group were 62% and 45% in the rhTPO group, and high‐risk groups were reported in 64% and 56%, respectively. The key baseline and clinical characteristics are summarized in Table 1.

**TABLE 1:** Baseline demographic and clinical characteristics of the patients

Characteristic	Eltrombopag (*n* = 44)	rhTPO (*n* = 48)	*p*‐Value
Recipient age at HSCT, median (range), yr	36 (18–54)	37 (18–57)	0.997
≥45 years, *n* (%)	8 (18)	14 (29)	
Recipient sex, *n* (%)			
Male	29 (66)	25 (52)	
Female	15 (34)	23 (48)	0.179
Underlying disease, *n* (%)			
AML	29 (62)	23 (45)	
ALL	13 (28)	19 (37)	0.186
MDS	2 (4)	6 (12)	
Disease status at HSCT, *n* (%)			
Standard risk	16 (36)	21 (44)	
High risk	28 (64)	27 (56)	0.470
Disease remission status, *n* (%)			
CR1	33 (75)	35 (73)	
CR2	7 (16)	7 (15)	0.886
NR	4 (9)	6 (12)	
ABO compatibility, *n* (%)			
Match	27 (61)	28 (58)	
Mismatch	17 (39)	20 (42)	0.767
Donor type, *n* (%)			
Total HLA‐matched sibling	11 (25)	14 (29)	
HLA‐mismatched sibling or parent	30 (68)	32 (67)	0.770
HLA‐matched unrelated donors	3 (7)	2 (4)	
Donor age, yr., median (range)	33 (10–61)	38 (12–64)	0.515
Donor/recipient sex match			
Male‐male	20 (45)	20 (41)	
Male‐female	10 (23)	7 (15)	0.395
Female‐female	4 (9)	10 (21)	
Female‐male	10 (23)	11 (23)	
Graft source G‐PB + G‐BM, median (range)			
PB‐CD34+cells (×10^6/kg)	6.21 (1.37–11.79)	5.53 (0.59–14.94)	0.866
BM‐CD34+cells (×10^6/kg)	0.84 (0.36–1.55)	0.72 (0.31–1.93)	0.774
Total‐CD34+cells (×10^6/kg)	6.21 (2.54–12.29)	5.64 (1.01–15.76)	0.708
PB‐ MNCs (×10^8/kg)	8.22 (5.5–15.42)	7.70 (3.35–18.72)	0.711
BM‐ MNCs (×10^8/kg)	2.80 (0.52–9.66)	3.17 (0.58–7.12)	0.384
Total MNCs (×10^8/kg)	10.53 (5.83–17.82)	10.73 (6.16–24.56)	0.808
Conditioning regime, *n* (%)			
BuCy	3 (7)	8 (17)	0.203
BuCy + ATG	41 (93)	40 (83)	

Abbreviations: ALL, acute lymphoblastic leukemia; AML, acute myeloid leukemia; ATG, antihuman thymocyte immunoglobulin; BM, Bone; BuCy, busulfan and cyclophosphamide; CR1, first complete remission; CR2, second complete remission; HSCT, hematopoietic stem cell transplantation; MDS, myelodysplastic syndromes; MNCs, monocytes; n, number; NR, none remission; PB, peripheral blood; rhTPO, recombinant human thrombopoietin.

### 3.2 Platelet engraftment and secondary endpoints

**FIGURE 2:**
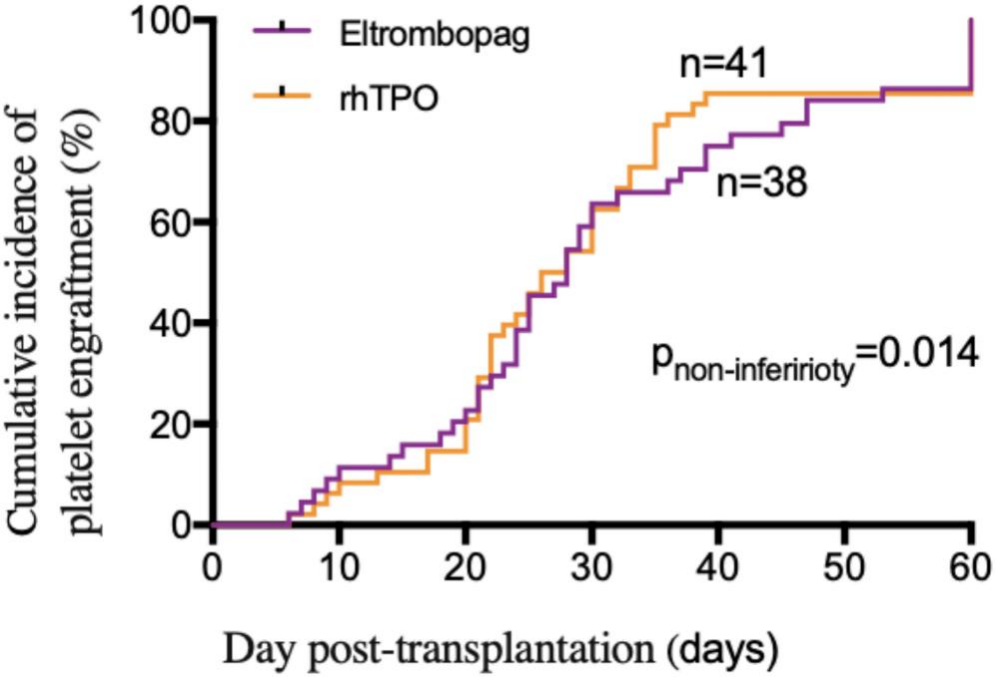
The cumulative incidence of platelet engraftment on day 60 post transplantation in eltrombopag and eltrombopag or recombinant human thrombopoietin (rhTPO) groups

**FIGURE 3:**
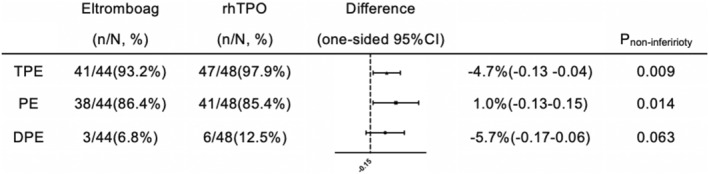
The cumulative incidence of total platelet engraftment, platelet engraftment on day 60, and Delayed platelet engraftment (DPE) post transplantation in eltrombopag and eltrombopag or recombinant human thrombopoietin (rhTPO) groups

41 patients (41/44, 93.2%) in eltrombopag group and 47 patients (47/48, 97.9%) in rhTPO group obtained platelet engraftment, meeting the non‐ferior criteria (absolute risk difference [ARD] −4.7%, one‐sided lower limit of 95%CI −13.21%, P_non‐inferirioty_ = 0.009). Median duration of follow‐up was 360 days (range: 12–960 days). The cumulative incidence of platelet engraftment on day 60 after transplantation in the eltrombopag group was 86.4% (38/44) compared with 85.4% (41/48) in the rhTPO group, meeting the non‐ferior criteria (ARD 1%, one‐sided lower limit of 95% CI −13.28%, P_non‐inferirioty_ = 0.014) (Figure 2). The rate of DPE in the eltrombopag group was 6.8% (3/44) compared with 12.5% (6/48) in the rhTPO group (ARD −5.7%, one‐sided higher limit of 95% CI 6.28%, P_non‐inferirioty_ = 0.063) (Figure 3).

**FIGURE 4:**
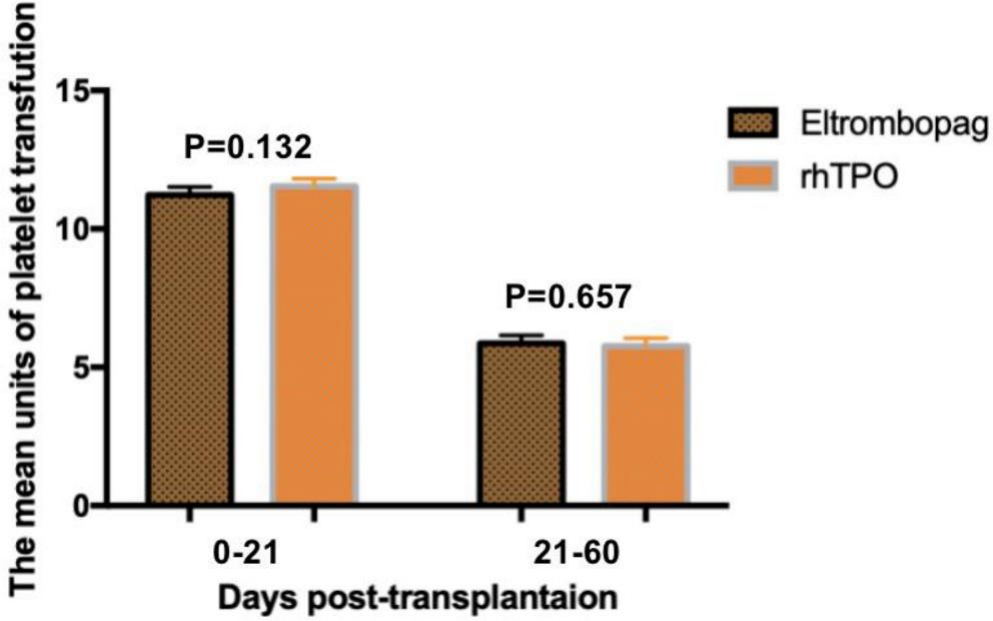
The mean units of platelets transfusion from day 0 to days 21, day 21 to days 60 post transplantation in eltrombopag and eltrombopag or recombinant human thrombopoietin (rhTPO) groups

The mean of platelet transfusion units from day 0 to day 21 was 11.23 ± 0.15 U (95% CI, 10.93–11.52) in the eltrombopag group and 11.54 ± 0.14 U (95% CI, 11.26–11.82) in the rhTPO group (*P* = 0.132), and the mean units of platelet transfusion from day 21 to day 60 were 5.86 ± 0.15 U (95% CI, 5.57–6.12) and 5.77 ± 0.14 U (95% CI, 5.49–6.05) in the eltrombopag and rhTPO groups, respectively (*P* = 0.657) (Figure 4).

All the patients underwent neutrophil and white blood cell engraftment. Median time of neutrophil engraftment was not statistically different between both groups: 18 days (range: 11–27 days) in eltrombopag group and 18 days (range: 11–31 days) in rhTPO group (*P* = 0.217).

### 3.3 Safety analysis and transplant‐related outcomes

**FIGURE 5:**
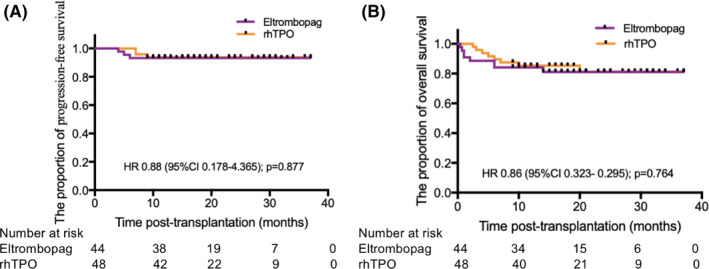
Progression‐free survival (PFS) and overall survival (OS) in eltrombopag and eltrombopag or recombinant human thrombopoietin (rhTPO) groups

One patient in eltrombopag group and four patients in rhTPO group died of respiratory failure. Two patients in eltrombopag group and four patients in rhTPO group died of grade 4 a‐GVHD with nervous system disease or severe gastrointestinal hemorrhage. In addition, two patients in eltrombopag group and none in rhTPO group died due to relapse. The probability of PFS was not statistically different between eltrombopag and rhTPO groups (HR 0.88; 95%CI 0.178–4.365; *p* = 0.877), and the probability of OS was also not statistically different between eltrombopag and rhTPO groups (HR 0.86; 95%CI 0.323–0.295; *p* = 0.764) (Figure 5).

Adverse events involving renal, hepatic, or coagulative functions were not statistically different between eltrombopag and rhTPO groups. Two patients in eltrombopag group and one patient in rhTPO group had gastrointestinal tract bleeding within 60 days after transplantation (*P* = 0.737), and other bleeding events, including oral mucositis and urinary bladder bleeding, were not statistically different between two groups (Table 2).

**TABLE 2:** Adverse events (AEs), Bleeding Events, Viremia and a‐GVHD

	Eltrombopag (*n* = 44)		rhTPO (*n* = 48)		*p*‐value
Laboratory abnormalities, *n* (%)	Grade half	Grade three‐fourths	Grade half	Grade three‐fourths	
^a^Hepatic function abnormalities	40 (91)	0 (0)	43 (90)	2 (4)	0.545
^b^Renal function abnormalities	17 (40)	0 (0)	21 (43)	1 (2)	0.750
^c^Coagulation abnormalities	5 (11)	0 (0)	2 (4)	0 (0)	0.253
^d^Total laboratory abnormalities	44 (100)	0 (0)	45 (100)	3 (6)	0.243
Bleeding events, *n* (%)					
Oral mucositis	1 (2)	23 (52)	3 (6)	19 (40)	0.366
Urinary bladder bleeding	4 (9)	14 (32)	5 (10)	20 (42)	0.560
GI tract bleeding	1 (2)	1 (2)	0 (0)	1 (2)	0.737
Viremia, *n* (%)					
EB viremia	27 (61)		38 (79)		0.061
MCV viremia	25 (57)		32 (67)		0.452
a‐GVHD, *n* (%)	26 (59)		33 (69)		0.335

Abbreviations: The adverse events were defined using the Common Terminology Criteria for Adverse Events, (CTCAE) version 4.0; a Hepatic function abnormalities included abnormalities in the values for glutamic‐pyruvic transaminase (ALT); glutamic‐oxaloacetic transaminase (AST); total bilirubin (TBIL); direct bilirubin (DBIL); bRenal function abnormalities included abnormalities in blood urea nitrogen(BUN); serum creatinine (Scr); *c* Coagulation abnormalities included abnormalities in the values for serum prothrombin time (PT); activated partial thromboplastin time (aPTT); fibrinogen (FIB‐C); D‐dimer; d Total laboratory abnormalities included abnormalities in all of the parameters mentioned above. EB, Epstein‐Barr; MCV, cytomegalovirus; a‐GVHD, acute graft‐versus‐host disease; *n*, number.

**FIGURE 6:**
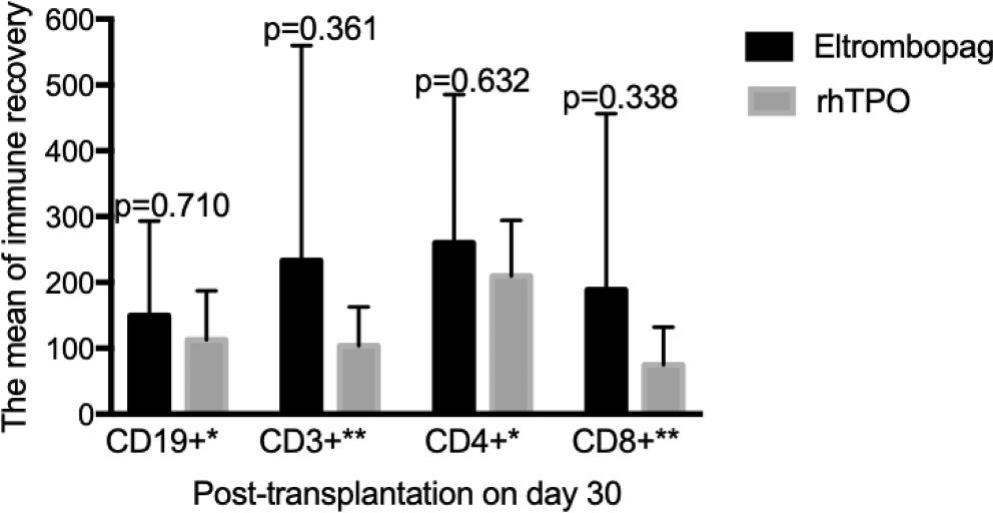
Immune recovery on day 30 post‐transplantation in eltrombopag and eltrombopag or recombinant human thrombopoietin (rhTPO) groups

None of the patients developed thrombotic or endothelial complications or hepatic veno‐occlusive disease in the study. No myelofibrosis was observed in this study. In addition, the rate of transplant‐related outcomes, including EBV and CMV infection within 100 days, and a‐GVHD did not significantly differ between eltrombopag and rhTPO groups (Table 2). Similarly, immune recovery was also not significantly different between both groups on day 30 post‐transplantation (Figure 6).

## 4 DISCUSSION

Delayed platelet implantation after allo‐HSCT is a common problem, and its morbidity is associated with increased patient mortality. Currently, platelet‐increasing drugs used after transplantation include eltrombopag and rhTPO. In this study, eltrombopag was non‐inferior to rhTPO in promoting platelet engraftment on day 60 post transplantation. Similarly, DPE, PFS, and OS were not statistically different between the two groups.

Luigi J. et. al. Showed that eltrombopag mediated the pro‐inflammatory cytokine interferon‐γ (IFNγ) to maintain human hematopoietic stem and progenitor cells under inflammatory conditions.^26^ Ahmed et. al. in 2021 showed that the eltrombopag group (starting on day 35 after transplantation, 8‐week continuous treatment) effectively promoted platelet recovery (≥50 × 10^9^/L) compared with the placebo group (9/42, 21% and 0/18, 0%, respectively; *P* = 0.046).^40^ Additionally, Ting‐ting Ha et. al. in 2014 showed that the cumulative platelet implantation rate (≥20 × 10^9^/L) in the rhTPO group within 60 days after transplantation was better than that in the placebo group (*P* = 0.041).^20^ In this study, eltrombopag was non‐inferior to rhTPO in promoting platelet engraftment on day 60 post transplantation. DPE, PFS, and OS were not signficantly different between the two groups after transplantation. Eltrombopag was non‐inferior to rhTPO in promoting platelet engraftment, and both eltrombopag and rhTPO decreased the number of platelet transfusions post‐transplantation. However, previous studies showed that some patients with rhTPO treatment might experience a secondary reduction in platelets due to the neutralizing autoantibodies during follow‐up.^41^, ^42^, ^43^ Therefore, eltrombopag, as a thrombopoietin‐receptor agonists, might be recommended firstly for promting platelet engraftment after allo‐HSCT with safety and effectiveness. Further studies are required to evaluate the mechanism of eltrombopag versus rhTPO after HSCT. Furthermore, oral eltrombopag is more convenient for patients than subcutaneous rhTPO.

In this study, eltrombopag and rhTPO administered to patients post‐transplantation were well tolerated, which is consistent with the conclusions of previous studies.^18^, ^44^ No significant difference was reported in the incidence of AEs, including bleeding and laboratory abnormalities, between the eltrombopag and rhTPO groups. In addition, none of the patient in the two groups died due to bleeding events, and no statistical difference was assessed in OS and PFS between eltrombopag and rhTPO groups. Akira H. et. al. In 2016 showed that the development of myelofibrosis during eltrombopag treatment was observed in a patient with immune thrombocytopenia.^45^ However, neither myelofibrosis nor clone evolution was found in this study during eltrombopag treatment. Therefore, eltrombopag was safe for promoting platelet recovery.

The mechanism of thrombocytopenia after allo‐HSCT is complex and affected by many factors, including injected stem cell counts, stem cell source, CMV serum status, donor type, HLA compatibility, a‐GVHD, ABO compatibility, intensity of pretreatment regimen, T cell depletion in the body, infections and medications (e.g., ganciclovir). This study found that no statistical difference in the rate of a‐GVHD or virus‐related infections between the two groups, which is consistent with the results of Ahmed et. al.^40^

There are some limitations in this trial. Due to small number of participants enrolled, it was difficult to analyzed in the subgroup, and the results may be biased. In addition, this trial was performed at a single center, and potential mechanisms are not yet settled underlying these results. Further studies are required to explore the mechanism of eltrombopag versus rhTPO after HSCT.

## 5 CONCLUSIONS

Eltrombopag was non‐inferior to rhTPO in promoting platelet engraftment after allo‐HSCT for patients with hematological malignancy. Oral eltrombopag was more convenient for patients than subcutaneous rhTPO.

## CONFLICT OF INTEREST

The authors declare that they have no competing interests.

## AUTHOR CONTRIBUTION

Xin Du and Qingli Gu contributed to conception and design of the study. Xiaohan Zhang, Siyu Chen, Jingchao Fan, Sitian Yang, Yun Cai, Pengcheng Wang and Qiaoxia Zhang contributed to acquisition of data. Bingbing Wen performed analysis of data and drafted the manuscript. All authors contributed to interpretation of the results and revised the manuscript. All authors read and approved the final manuscript.

### TRANSPARENT PEER REVIEW

The peer review history for this article is available at https://publons.com/publon/10.1002/hon.3017

## Data Availability

The data that support the findings of this study are available on request from the corresponding author. The data are not publicly available due to privacy or ethical restrictions.
